# Commentary: Integrating gamified learning to support competency-based nursing supervision

**DOI:** 10.1177/17449871251391150

**Published:** 2026-01-18

**Authors:** Sarah Bekaert

**Affiliations:** Associate Professor, Oxford School of Nursing and Midwifery, Oxford Brookes University, Oxford, UK

**Commentary to:** Enhancing clinical supervision in nursing through the SUPERVISE^®^ serious game: validation and usability study (Fernandes et al., 2025).

Practical training in clinical settings is essential for nursing education, with the quality of clinical supervision directly impacting student learning outcomes. Supervisors play a central role in guiding students, yet challenges such as limited time, high workload, and insufficient feedback skills often reduce supervision quality. To address these challenges, innovative strategies such as gamification have been adopted in healthcare education, improving engagement, motivation, knowledge acquisition, skill development and confidence building (Fusco, 2022; Krishnamoorthy, 2025). The SUPERVISE^®^ serious game was developed as a theory-informed intervention to enhance clinical supervision training in nursing. This study aimed to validate the content of SUPERVISE^®^ and assess its usability among nurses.

A mixed-methods approach was employed, conducted between November 2023 and July 2024. The study consisted of two phases: the validation of SUPERVISE^®^ game content via a modified e-Delphi method and usability assessment with practising nurses. Thirty-six experts in clinical supervision and nursing education were recruited through purposive and snowball sampling to validate content across six thematic categories. The Delphi process involved iterative rounds in which experts refined items until consensus and stability were reached. Only items achieving ⩾85% consensus and demonstrating stability were included in the final game content (Bownes and Giannotti, 2023; Fernandes and Magalhães, 2024).

In the first e-Delphi round, 45 experts (23.9% response rate) reviewed 128 items, reaching 96% consensus. Seventy-seven items required revision for round two, in which 36 experts (80% retention) participated. The second round achieved 97.9% consensus, confirming the robustness and relevance of the game content. This process ensured that SUPERVISE^®^ reflects evidence-based and academically rigorous clinical supervision principles, providing a solid foundation for training nurses in practice (Landers, 2020; Wijnen-Meijer et al., 2022).

Usability was assessed with 39 nurses using the Portuguese version of the System Usability Scale (SUS; Martins et al., 2015) and the Serious Educational Game in Nursing Appraisal Scale (SEGiNAS; Fernandes et al., 2024), complemented by open-ended feedback. The game achieved an SUS score of 79.4, indicating good usability. Participants highlighted its playful, reflective and interactive elements, whereas minor limitations included duration, accessibility, and clarity of instructions. These findings suggest that SUPERVISE^®^ is a feasible and acceptable tool for clinical supervision training, capable of fostering engagement, reflective practice, and practical skill development (Garmy and Bekaert, 2025).

SUPERVISE^®^ is built on strong learning theory foundations that are frequently used in nursing education, ensuring that its design supports not only knowledge acquisition but also motivation, reflection, and self-efficacy. Kolb’s Experiential Learning Cycle emphasises the integration of experience, theory, and simulation to support practice-based learning (Kolb, 1984). For example, Wijnen-Meijer et al. (2022) demonstrated how integrating Kolb’s cycle into nursing education through a combination of theory, simulation and practice enhances active, practice-based learning and skill development. Fernandes et al. (2025) also drew on Self-Determination Theory (Ryan and Deci, 2000), which highlights the role of autonomy, competence and relatedness in motivating healthcare professionals to engage with digital learning tools (Ross, 2018). Furthermore, constructivist learning approaches (Piaget, 1973; Vygotsky, 1978) focus on active knowledge construction, enabling supervisors to develop critical thinking and cultural competence; for example, Hunter (2008) examined the use of constructivist approaches in nursing education, showing how actively engaging students in knowledge construction develops critical thinking and enhances cultural competence in clinical practice. Finally, Bandura’s Social Cognitive Theory (Bandura, 1986) underpins clinical learning by emphasising self-efficacy, observational learning and feedback, reinforcing the importance of modelling supervisory behaviours. Similarly, Landers (2020) applied Bandura’s theory to nursing education, demonstrating how structured clinical learning experiences that incorporate modelling, feedback and observational learning enhance students’ self-efficacy and skill development. Collectively, these theories position SUPERVISE^®^ as more than a digital game; it is a theoretically informed intervention that strengthens the conceptual foundations of nursing supervision.

The validation of SUPERVISE^®^ demonstrates its potential as an evidence-based tool for integration into nursing curricula and continuing professional development. Serious games (designed for purposes beyond pure entertainment, such as education, training, health, or social change) and digital learning platforms have been recognised as strategies to support competency-based training and innovative workforce development (Bekaert et al., 2025). By setting standards for digital pedagogical tools, SUPERVISE^®^ can inform institutional and national frameworks for nurse training, supporting structured clinical supervision, and promoting reflective practice among supervisors.

In my recent commentary, *Repositioning health education as a core nursing function* (Bekaert, 2025), I argued that educating students and peers is a central responsibility of nurses, highlighting the importance of structured supervision and reflective practice. This perspective aligns directly with the design and purpose of SUPERVISE^®^: by providing structured, interactive digital scenarios, furthermore the game creates opportunities for supervisors to consciously reflect on their personal and professional values while guiding students. Similarly, our Editorial *What is nursing to you? The importance of personal values in nursing practice* (Garmy and Bekaert, 2025) underscores that reflective engagement with personal values informs decision-making, professional behaviour, and interactions with patients – principles embedded in SUPERVISE^®^’s reflective tasks. Together, these reinforce the idea that structured digital tools can operationalise reflective practice, enabling supervisors to integrate their values into clinical supervision and supporting high-quality, consistent nursing education at both institutional and national levels. SUPERVISE^®^ offers a scalable, evidence-based model for enhancing nursing education and professional development through gamified, competency-focused approaches.
